# Health Tract No. 324

**Published:** 1868-05

**Authors:** 


HEALTH TRACT No. 324.

### A 3000 DOLLAR BED!

Such was the price paid by a lady friend of ours a few days ago, and does not the reader exclaim with impatience; "what are we coming to"? "where is all this extravagance to end"? Let us look at the particulars, bearing in mind a self evident truth, that "the case being altered, alters the case." It was the Lady's own money; amassed by judicious economies; by persistent self denials; it really was a very fine bed; she had something to show for her money; and that is more than ye wine bibbing, smoking, chewing, tea drinking folk can say; and many of you did'nt earn your three thousand, it was left to you; or you got, it may-hap, by turning that short corner, or by some little smart trick, that the law could not reach and men called it a "business transaction." The bed we speak of is a very substantial one; and it is already put up in a building, easily seen from our house, so there is no mistake about the price, or its locality, or its quality. Besides being costly, and substantial, it has a commendable peculiarity; it can never be taken down; and it will be always in use; and long after this stirling hearted widow has been sleeping beside her noble husband and "little Tommy" in the quiet church yard, near where she spent the sunny days of childhood, this bed will be one of "down," and will never wear out; and men and women too, will bless the name and memory of her who made the purchase; and six other persons in New York paid the same price each, for six other beds; during 1867; and before that, forty-eight other beds had been bought at the same price, and all put up in the same house; built by the enterprize and the money, in great part, of him who wrote the immortal hymn,

"I would not live alway."

These fifty-five beds are in the St. Luke Hospital, on Fifth Avenue, New York; for the benefit of those who are sick and poor and have no home; nor any friend who can give them a helping hand. Every three thousand dollars paid, is put out at safe and sure interest, and the income of it pays for the board, bed, washing, medical attendance and everything else that money can needfully procure, for any human being, young or old, catholic or protestant who can pay nothing: on recovery or death, this bed is ready for the first other unfortunate who applies; and so it will be while our country stands. What a noble heart it was reader, that saved and paid, three thousand dollars for a bed!" Bless the women, bless the widows, who thus deny themselves to bless others! others whom they never knew, but who bear the image of Him who died for them, and washed them in his blood.

